# A comprehensive assessment of impulsivity requires more than a single questionnaire

**DOI:** 10.47626/2237-6089-2023-0678

**Published:** 2024-11-06

**Authors:** Isabella Fonseca, Carl Hart

**Affiliations:** 1 Columbia University Barnard College New York NY USA Barnard College, Columbia University, New York, NY, USA.

I read with interest the article by Wagner et al.^1^ that sought to investigate impulsivity in people diagnosed with cannabis use disorder. It was found that roughly a third of participants endorsed a total number of symptoms that reached deficit levels, as operationally defined by the investigators. This finding prompted the researcher to conclude that they found the "presence of impulsive behavior among individuals with cannabis use disorder." At least one issue warrants further discussion because this conclusion extends beyond the study's data and its inherent limitations.

Only one instrument – the Barratt Impulsiveness Scale (BIS-11) – was used to assess impulsivity, which by nature is a multifaceted construct comprised largely of complex behavioral acts. The BIS-11 was not designed to measure "impulsive *behavior*," yet this is what is discussed throughout the paper and in the conclusion. The BIS-11 is a questionnaire composed of 30 items, to which respondents answer on a four-point Likert scale of 1 = Rarely/Never to 4 = Almost Always/Always. For example, the items "I like puzzles?" and "I like to think about complex problems" are included in the questionnaire. In other words, the BIS-11 is a self-report measure of only *verbal* recognition of specific behaviors and does not demonstrate any observable or *actual* behaviors.

Without the inclusion of additional assessment batteries, it would be inappropriate to draw conclusions about cannabis's influence on impulsivity. It would have helped to have included a variety of measures that tapped overlapping and diverging aspects of impulsivity. There are many widely accepted measures that tap different components of impulsivity and using them in combination is the best way of attempting to understand complex behaviors such as impulsivity. Providing multiple measures to test for impulsivity, especially observing cognitive behavioral tasks, would have increased our confidence in the study's results.

Given the above concern, it is unlikely that this study tapped the complex nature of impulsivity. Therefore, the study's conclusion is misleading because they extend far beyond the confines of the data. While the researchers briefly state in their conclusion that their study does not provide a platform for the cause-and-effect relationship to be drawn, they do strongly suggest a correlative relationship between impulsivity and cannabis use disorder based on the singular measure used. The implication of such a study that only uses one measure to test impulsivity is vast; specifically, it provides misleading information to people in society in regard to cannabis use. It is my hope that future studies employ measures that would ensure a more comprehensive assessment of impulsivity in people with cannabis use disorders.
